# Receiving or not deemed necessary healthcare services

**DOI:** 10.1186/s12889-023-15135-7

**Published:** 2023-01-31

**Authors:** Seher Nur Sulku, Yagmur Tokatlioglu, Kubra Cosar

**Affiliations:** Department of Econometrics, Ankara Haci Bayram Veli University, Muammer Bostanci Caddesi No: 4, 06500 Ankara, Türkiye Turkey

**Keywords:** Healthcare Avoidance, Tertiary Care, Characteristic and reasons of Avoiders, Bivariate Probit Model, Türkiye

## Abstract

**Background:**

Avoiding deemed necessary healthcare needs may worsen prognosis and treatment options, and damage people’s ability to perform their roles in society. Our study investigates why people avoid healthcare services in an upper-middle-income country, Türkiye.

**Methods:**

We apply TurkStat’s 2012 Health Survey Data that includes a comprehensive health and social-demographic information of 28,055 survey participants who were 15 + aged. We use bivariate probit model to analyze the avoidance behavior in inpatient level in accordance with outpatient level because of the observed significant correlation between people’s avoidance behavior under tertiary and lower level health care.

**Results:**

The findings show that 2.6% of 15 + aged population avoided deemed necessary hospital services. Furthermore, we found that high cost (31%), organizational factors (21%) and fear (12%) are prominent reasons of avoiding tertiary care. Thereafter, in our bivariate probit model findings, we figure out that being covered by social security schemes decreases the probability of avoiding both outpatient and inpatient health services by 6.9%. Moreover, being female, living in rural area, having lower income increase the chance of being avoider in both stages of healthcare.

**Conclusion:**

We conclude that social inequalities are the main underlying determinants of the avoiding behavior.

## Background

Medical care avoidance is such a paradox that actually people could save their lives or ameliorate suffering. People all over the world avoid health care services for some reasons and this may lead to worsen prognosis and treatment options. Late diagnosis of diseases requires radical treatment and can lead to irreversible conclusions or even death [1–3]. In addition to negative effects on the health and treatment process, it has a financial burden on the health system and household budget. When treatments are carried out at the advanced stage of the disease, expenses for the recovery become higher compared to the earlier diagnosis due to the requirements for more radical treatments, longer surgery time and hospital stay [2, 4]. Regarding adverse effects on human health and economy of medical care, avoidance behavior is taking attentions in the literature. [1, 4, 5] investigate the general reasons of avoiding, and [1, 6–8] explores socio economic determinants of avoiding. In addition, there are numerous studies investigating avoidance of seeking medical care behavior from gender perspective [9–11], from urban/rural area differences [12, 13], from age perspective [14–17]. There are also various studies searching avoidance factors from the specific diseases perspective such as chronic diseases [18], heart diseases [10, 19–23], cancer [24], diabetes [25], stroke [22], breast cancer [26–28], HIV [29, 30], tuberculosis [31, 32], Parkinson [33], mental health [34–36], osteoarthritis [37].

Our study explores the determinants of avoiding deemed necessary healthcare services in Türkiye, an upper middle-income country. In Türkiye, there have been health system reforms called “Health Transformation Programme” (*HTP*) since 2003. Overall, these reforms improve the accessibility and mitigating inequalities as universal health insurance was introduced and social security intuition’s (*SSI*) coverage of services have been expanded remarkably [38]. Indeed, since the noticeable progress in the health outcomes and the health system, the *HTP* was pointed out as a successful good practice example [39]. Though access to medical care services is necessary but not sufficient, people’s avoidance behavior should still be explored and since mentioned bench of reforms, Türkiye is an interesting example for other countries as well. In literature, we commonly observe that disadvantaged groups-like women, undereducated, the ones living in rural, poors etc. are more likely to face with barriers to reach health services, and thus they avoid necessary health care services [1, 2, 22, 40, 41]. Consequently, the hypothesis of this study is *H*_*0*_: Disadvantaged groups are more tend to avoid health care services. Furthermore, avoidance in seeking medical care may happen in different stages of health care provision [42]. Therefore, our study explores the determinants of avoiding healthcare services in hospitalization stage in accordance with outpatient services’ avoidance by applying bivariate probit model.

Our study is organized as follows: Under methodology section, we introduced our data, which is the Turkish Statistical Institute (TurkStat)’s health survey data 2012, and then established the methodology of bivariate probit regression models. Thereafter in the results section, first basic descriptive of our data set is given as compared the avoiders versus non-avoiders in the study examining the characteristics of participants who received or did not receive health care service even though treatment is recommended by doctor, either as an inpatient or a day patient (less than 24 h) in the last 12 months. Next, the reasons of avoidance behavior in tertiary care are briefly introduced. Thereafter the findings from the bivariate probit regression models are presented. In the discussion section, our findings are assessed in line with the existing literature, and limitations are stated. Finally, we conclude our study.

## Methods

### Data source

In this study, Health Survey, conducted by TurkStat for the first time in 2012, has been used as data source. The 2012 Health Survey was conducted with 14,400 households with 84.44% response rate. The rural-urban stratification was done considering 3,744 households from rural and 10,656 households from urban (with 83.78% and 86.32% response rates respectively). Totally 37,979 individuals’ information has been collected, 28,055 of them were aged at least 15 years old, 15+. Health Survey 2012’s study design is given in detail in TurkStat [43].

In our study, first we compared the characteristics of avoiders versus non-avoiders of healthcare at hospital utilization stage. Thus, the answers for the question coded S074000000, in Part 10 of Part B of the survey, were analyzed considering hospital services, asking participants whether she/he had not received health care service even though treatment recommended by doctor, either as an inpatient or a day patient in the last 12 months.

### The bivariate probit model

In Türkiye, as similar to other developing countries experiences [44, 45] the referral system does not work efficiently, and indeed significant correlation between the avoidance behavior in accessing outpatient level and hospitalization level services has been observed, thus we apply bivariate probit model to analyze the avoidance behavior in inpatient level in accordance with outpatient level usage of services. Now, we define people’s decision of avoiding or receiving outpatient care and inpatient care as the two dependent variables in the bivariate probit model and present them respectively as ${y}_{1}$ and ${y}_{2}$ as given below:

$${y_1} = \left\{ {1,\,avoid,\,outpatient\,0,\,not\,avoid,\,outpatient} \right.$$

$${y}_{2}=\{1,\hspace{0.25em}\hspace{0.25em}\hspace{0.25em}\hspace{0.25em} avoid, inpatient\ 0,\hspace{0.25em}\hspace{0.25em}\hspace{0.25em}\hspace{0.25em} not avoid, inpatient$$

here the first dependent variable, ${y}_{1}$, takes 1 if the individual avoids medical care deemed necessary in outpatient level, and the second dependent variable, y_2_, takes 1 if the individual avoids necessary medical care in inpatient level.

A natural extension of the probit model, bivariate probit model would allow more than one equation considering the correlation between error terms, as the seemingly unrelated regressions model. The general structure of a two-equation bivariate probit model is as follows [46]:

$${y}_{1}^{*}={x}_{1}^{{\prime }}{\beta }_{1}+{\epsilon }_{1}, {y}_{1}=1 if {y}_{1}^{*}>0,\ 0\ otherwise$$

$${y}_{2}^{*}={x}_{2}^{{\prime }}{\beta }_{2}+{\epsilon }_{2}, {y}_{2}=1 if {y}_{2}^{*}>0 , 0\ otherwise$$

here $${y}_{1}^{*}$$ and $${y}_{2}^{*}$$ are latent variables if they are positive then we can observe respectively y_1_ & y_2_, and $${x}_{1},{x}_{2}$$ are explanatory variables, and $${\epsilon }_{1}$$&$${\epsilon }_{2}$$ are error terms such that: $$E\left[{\epsilon }_{1}\mid {x}_{1},{x}_{2}\right]=E\left[{\epsilon }_{2}\mid {x}_{1},{x}_{2}\right]=0,$$$$Var\left[{\epsilon }_{1}\mid {x}_{1},{x}_{2}\right]=Var\left[{\epsilon }_{2}\mid {x}_{1},{x}_{2}\right]=1,$$$$Corr \left[{\epsilon }_{1},{\epsilon }_{2}\mid {x}_{1},{x}_{2}\right]=\rho$$. The bivariate probit model (1) introduces the correlation between error terms (rho:$$\rho$$) and concludes that the two-avoidance behavior shown by the two dependent variables are also correlative.

## Results

In this section, first of all we introduce the descriptive statistics analysis of characteristics of healthcare avoiders with respect to non-avoiders, and then visually figure out the reasons of avoidance. Thereafter cross distribution of avoidance behavior for outpatient and inpatient care is analyzed and since significant relationship was seen between them, the bivariate probit model is derived. Finally, the findings of the bivariate model are established.

### Descriptive statistics

Table 1 presents a comparison of the characteristics of avoiders and non-avoiders such as demographic features, social status and health conditions. In addition to the characteristics of avoiders and non-avoiders, ${\chi }^{2}$ test was applied for categorical variables and *t*-test was applied for continuous variables. As discussed by [47] these tests are very well known and commonly applied in empirical literature. First of all, 730 individuals constituting 2.6% of 15 + aged population were avoider and 27,244 individuals constituting 97.11% of 15 + aged population were non-avoider.^1^ When we look at gender, 32.88% of avoiders are male and 67.12% female versus 46.44% of non-avoiders are male and 53.56 are female (${\chi }^{2}$(1) = 52.61 and *p* < 0.001), thus avoiders are more likely to be female. Moreover, it is seen that avoiders more likely to be elderly, living rural area, married, low educated, having green card - public scheme for poor- and having lower income. Furthermore, working people and retired ones are less likely to avoid hospitalization stage, but avoiders are more likely to be occupied with house duties. Moreover, avoiders’ health conditions were worse when compared to non-avoiders, such as 80.27% of avoiders have any chronic diseases but only 50.46% of non-avoiders have and this difference is statistically significant (${\chi }^{2}$(1) = 268.61 and *p* < 0.001).

**Table 1:** Avoiders versus non avoiders: Characteristics

	Avoider (n = 730)	Non-Avoider (n = 27,244)	${\chi }^{2}$value, *p* value	
	n (%)	n (%)		
Gender			${\chi }^{2}$(1) = 52.61, *p* < 0.001	
Male	240(32.88%)	12,651 (46.44%)		
Female	490 (67.12%)	14,593 (53.56%)		
Age			${\chi }^{2}$ (6) = 63.67, *p* < 0.001	
15–24	71 (9.73%)	5036 (18.48%)	${\chi }^{2}$(1) = 36.548, *p* < 0.001	
25–34	119 (16.3%)	5471 (20.08%)	${\chi }^{2}$ (1) = 6.353, *p* = 0.012	
35–44	158 (21.64%)	5377 (19.74%)	${\chi }^{2}$ (1) = 1.629, *p* = 0.202	
45–54	153 (20.96%)	4760 (17.47%)	${\chi }^{2}$ (1) = 5.971, *p* = 0.015	
55–64	98 (13.42%)	3353 (12.31%)	${\chi }^{2}$ (1) = 0.821, *p* = 0.365	
65–74	79 (10.82%)	2029 (7.45%)	${\chi }^{2}$ (1) = 11.618,*p* = 0.001	
75+	52 (7.12%)	1218 (4.47%)	${\chi }^{2}$ (1) = 11.543,*p* = 0.001	
Location			${\chi }^{2}$ (1) = 31.127,*p* < 0.001	
Urban	472 (64.66%)	20,127 (73.88%)		
Rural	258 (35.34%)	7117 (26.12%)		
Marital status			${\chi }^{2}$ (3) = 83.298,*p* < 0.001	
Single	81 (11.1%)	6307 (23.15%)	${\chi }^{2}$ (1) = 58.625,*p* < 0.001	
Married	540 (73.97%)	18,624 (68.36%)	${\chi }^{2}$ (1) = 10.38, *p* = 0.001	
Widow	84 (11.51%)	1718 (6.31%)	${\chi }^{2}$ (1) = 31.909,*p* < 0.001	
Divorced	25 (3.42%)	595 (2.18%)	${\chi }^{2}$ (1) = 5.05, *p* = 0.025	
Education			${\chi }^{2}$ (4) = 77.278,*p* < 0.001	
Illiterate	135 (18.49%)	2750 (10.09%)	${\chi }^{2}$ (1) = 54.224,*p* < 0.001	
Primary school	416 (56.99%)	14,699 (53.95%)	${\chi }^{2}$ (1) = 2.633, *p* = 0.105	
Elementary school	37 (5.07%)	1643 (6.03%)	${\chi }^{2}$ (1) = 1.166, *p* = 0.280	
High school	86 (11.78%)	4841 (17.77%)	${\chi }^{2}$ (1) = 17.569,*p* < 0.001	
University and higher degree	56 (7.67%)	3311 (12.15%)	${\chi }^{2}$ (1) = 16.015 *p* < 0.001	
Estimated household monthly income			${\chi }^{2}$ (3) = 74.162,*p* < 0.001	
Less or equal to 750 TLǂ	242 (33.8%)	5679 (21.08%)	${\chi }^{2}$ (1) = 64.521,*p* < 0.001	
751 TL to 1300TL	218 (30.45%)	8383 (31.12%)	${\chi }^{2}$1) = 0.275, *p* = 0.600	
1301 TL to 2300TL	161 (22.49%)	7933 (29.45%)	${\chi }^{2}$ (1) = 17.251,*p* < 0.001	
Greater than 2300TL	95 (13.27%)	4939 (18.34%)	${\chi }^{2}$ (1) = 12.605,*p* < 0.001	
Working statue			${\chi }^{2}$ (5) = 96.88, *p* < 0.001	
Works	235 (32.19%)	10,188 (37.40%)	${\chi }^{2}$ (1) = 8.235, *p* = 0.004	
Looking for a job or seasonal worker	33 (4.52%)	1028 (3.77%)	${\chi }^{2}$ (1) = 1.088, *p* = 0.297	
Student	28 (3.84%)	2797 (10.27%)	${\chi }^{2}$ (1) = 32.385,*p* < 0.001	
Occupied with house duties	288 (39.45%)	8682 (31.87%)	${\chi }^{2}$ (1) = 18.774,*p* < 0.001	
Retired	61 (8.36%)	3011 (11.05%)	${\chi }^{2}$ (1) = 5.285, *p* = 0.022	
Other	85 (11.64%)	1538 (5.65%)	${\chi }^{2}$ (1) = 46.809,*p* < 0.001	
Heath insurance statue			${\chi }^{2}$ (3) = 79.408,*p* < 0.001	
SSI^*^	533 (73.01%)	22,802 (83.7%)	${\chi }^{2}$ (1) = 58.641,*p* < 0.001	
Green card^**^	122 (16.71%)	2385 (8.75%)	${\chi }^{2}$ (1) = 55.187,*p* < 0.001	
Private insurance	20 (2.74%)	916 (3.36%)	${\chi }^{2}$ (1) = 0.852, *p* = 0.356	
Out of pocket	55 (7.53%)	1141 (4.19%)	${\chi }^{2}$ (1) = 19.451,*p* < 0.001	
Perceived health			${\chi }^{2}$ (4) = 617.25,*p* < 0.001	
Very good	24 (3.29%)	3550 (13.03%)	${\chi }^{2}$ (1) = 60.557,*p* < 0.001	
Good	209 (28.63%)	15,301 (56.17%)	${\chi }^{2}$ (1) = 218.16,*p* < 0.001	
Fair	303 (41.51%)	6377 (23.41%)	${\chi }^{2}$ (1) = 128.13,*p* < 0.001	
Bad	156 (21.37%)	1786 (6.56%)	${\chi }^{2}$ (1) = 241.52,*p* < 0.001	
Very bad	38 (5.21%)	255 (0.83%)	${\chi }^{2}$ (1) = 146.42,*p* < 0.001	
Have a health problem more than 6 months			${\chi }^{2}$ (1) = 289.08,*p* < 0.001	
Yes	481 (65.98%)	9619 (35.33%)		
No	248 (34.02%)	17,608 (64.67%)		
Health problem restricts daily life			${\chi }^{2}$ (2) = 624.89,*p* < 0.001	
Seriously	257 (35.25%)	2673 (9.84%)	${\chi }^{2}$ (1) = 446.24,*p* < 0.001	
Not seriously	237 (32.51%)	5664 (20.85%)	${\chi }^{2}$ (1) = 58.23, *p* < 0.001	
Not restricted at all	235 (32.24%)	18.827 (69.31%)	${\chi }^{2}$(1) = 448.93,*p* < 0.001	
Feel pain or discomfort in last four weeks			${\chi }^{2}$ (1) = 535.76,*p* < 0.001	
Feel	402 (55.14%)	5412 (19.88%)		
Not Feel	327 (44.86%)	21,806 (80.12%)		
Feel happy in last four weeks			${\chi }^{2}$ (4) = 277.70,*p* < 0.001	
Always	74 (10.21%)	3718 (13.76%)	${\chi }^{2}$ (1) = 7.475, *p* = 0.006	
Most of the times	198 (27.31%)	12,146 (44.94%)	${\chi }^{2}$ (1) = 87.90, *p* < 0.001	
Sometimes	295 (40.69%)	9214 (34.09%)	${\chi }^{2}$ (1) = 13.763,*p* < 0.001	
Rarely	116 (16.00%)	1578 (5.84%)	${\chi }^{2}$ (1) = 127.44,*p* < 0.001	
Never	42 (5.79%)	369 (1.37%)	${\chi }^{2}$ (1) = 95.036,*p* < 0.001	
Number of persons to trust			${\chi }^{2}$ (1) = 20.955,*p* < 0.001	
Less or equal to 1	197 (26.99%)	5472 (20.09%)		
Greater or equal to 2	533 (73.01%)	21,772 (79.91%)		
Obese			${\chi }^{2}$ (1) = 21.268,*p* < 0.001	
Yes	180(24.66%)	4901(17.99%)		
No	550(75.34%)	22,343(82.01%)		
BMI^***^	28.617 (43.92)	27.718 (34.34)	*t*-test = 0.693,*p* = 0.4886	
Non-prescription medications in last two weeks			${\chi }^{2}$ (1) = 88.257,*p* < 0.001	
Yes	158 (21.64%)	2901 (10.65%)		
No	572 (78.36%)	24,343 (89.35%)		
Having any-chronic disease			${\chi }^{2}$(1) = 268.61, *p* < 0.001	
Yes	568 (80.27%)	13,497(50.46%)		
No	144(19.73%)	13,747 (50.54%)		
Hypertension			${\chi }^{2}$ (1) = 50.092,*p* < 0.001	
Yes	182 (24.93%)	4171 (15.31%)		
No	548 (75.07%)	23,073 (84.69%)		
Back musculoskeletal system disorders			${\chi }^{2}$ (1) = 360.38,*p* < 0.001	
Yes	272 (37.26%)	3515 (12.9%)		
No	458 (62.74%)	23,729 (87.1%)		
Rheumatismal joint disease			${\chi }^{2}$ (1) = 243.95,*p* < 0.001	
Yes	197 (26.99%)	2777 (9.93%)		
No	533 (73.01%)	25,197 (90.07%)		
Gastric ulcer			${\chi }^{2}$ (1) = 203.78,*p* < 0.001	
Yes	165 (22.6%)	2144 (7.87%)		
No	565 (77.4%)	25,100 (92.13%)		
Diabetes			${\chi }^{2}$ (1) = 16.74,*p* < 0.001	
Yes	87 (11.92%)	2120 (7.78%)		
No	643 (88.08%)	25,124 (92.22%)		
Cancer			${\chi }^{2}$1) = 6.173, *p* = 0.013	
Yes	11 (1.51%)	194 (0.71%)		
No	719 (98.5%)	27,050 (99.29%)		
Having mental health problems			${\chi }^{2}$ (1) = 164.75,*p* < 0.001	
Yes	102 (13.97%)	1123(4.12%)		
No	628 (86.03%)	26,121 (95.882%)		

ǂTL: Turkish Lira, *SSI : Social Security Institute, **Green Card a security scheme aim to cover the poor individuals to receive health services, ^***^Body Mass Index. If *BMI* < 18.5 underweight, 18.5 ≤ *BMI* ≤ 24.9 normal,25 ≤ *BMI* ≤ 29.9 overweight and *BMI* ≥ 30 obesity

### Reasons of avoidance

**Fig. 1:**
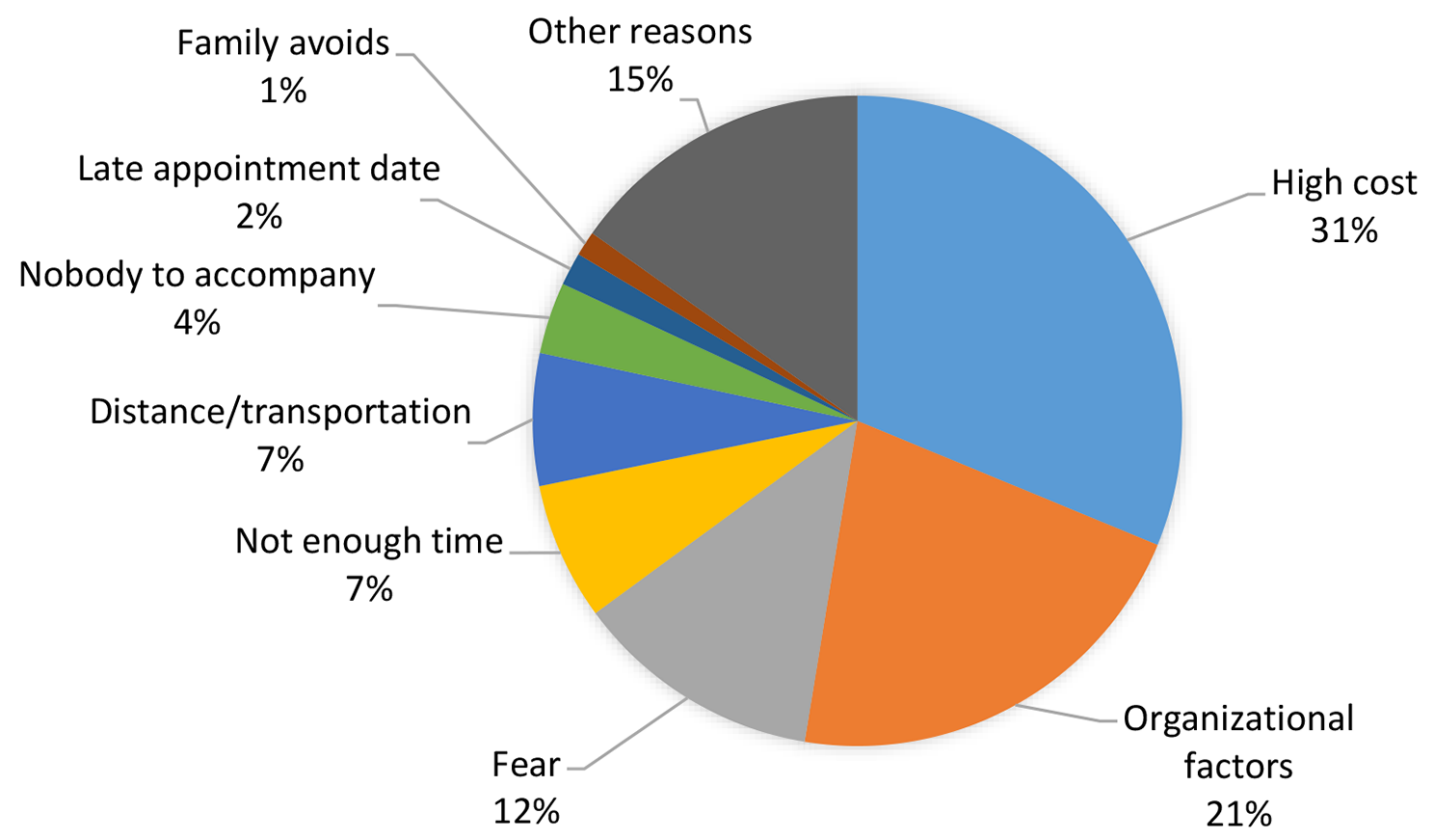
Distribution of the reasons of avoidance (%)

The 2012 health survey of TurkStat asks the avoiders to select one of the eight pre-identified reasons. As seen from Fig. 1, the main reason of avoiding hospitalization in Türkiye is high cost. Almost one third of the participants refrain from hospitalization since they cannot afford health services because of expensive prices or nonpayment by insurance. Organizational factors follow the high costs. 21% of the participants have pointed out that they avoid hospitalization because of difficulties in achieving treatment at polyclinics and other reasons related to health organization. Fear of medical treatment or surgery appears as the third important reason as 12% of respondents have indicated it as the major cause of avoidance. Consequently, all these eight reasons together explain 85% of the avoidance behavior; the remaining participants (15%) have marked other reasons option.

### Cross distribution of avoidance behavior under outpatient and inpatient care

Since the avoidance may occur at different stages of healthcare provision, we would like to see in which stage of utilization people are more likely to avoid. Our study finds that 2.6% of the 15 + aged participants were avoider in the hospitalization stage, while [48] study which used the same survey indicated that 12.6% of the 15 + aged participants were avoider for the primary healthcare services and/or outpatient services. We may say that 10%-point decrease in avoidance in hospitalization stage is not surprising since individuals use hospital services for more serious illness,.

Furthermore, we investigated the cross distribution of avoiders and non-avoiders under primary and/or outpatient levels versus hospitalization level services. Behavior of avoiders for the primary healthcare services and/or outpatient services (to call it briefly as ‘first services’) and hospitalization is investigated more detailed in Table 2. Among all participants, 480 individuals were avoiders under both level of services and 24,126 individuals were non-avoiders under both level of services. Thus, we see that in total 88.23% (=(480 + 24,126)/27,889) of participants did not change their attitude of avoiding or receiving under different stages of health utilization.

**Table 2:** Crosstab of primary/outpatient services and hospitalization

		Primary healthcare services and/or outpatient services		
		Avoiders	Non-Avoiders	Total
**Hospitalization**	**Avoiders**	480	249	729
	**Non-Avoiders**	3034	24,126	27,160
	**Total**	3514	24,375	27,889

### The bivariate probit model findings

Because of the observed relationship (in Table 2) between the avoidance/non-avoidance behavior in accessing outpatient level and hospitalization level services, the bivariate probit model is employed to examine the avoidance behavior in inpatient level in accordance with outpatient level usage of services.

It should be noted that the bivariate probit model has been applied on a sample of 12,032 respondents aged 15 years old or older which was randomly taken from 2012 Health Survey. As it may be seen that the correlation between the avoidance outpatient services and inpatient services is high in Table 3, being *rho = 0.5661* and significant. It means that people who avoid/non-avoid visiting doctor for outpatient are more likely to avoid/non-avoid inpatient care services. Especially since Health Transformation Programme in 2003–2012, some legislations have been carried out in order to implement the referral system in Türkiye. However, due to the health system and social factors, they are not implemented [49]. Considering both the high correlation coefficient and status of the referral system in Türkiye, it is convenient to use the bivariate probit model to estimate behavior of avoidances.

First, we have analyzed determinants of avoidance by using bivariate probit model. Then, marginal effects are estimated across the four joint probabilities: avoiding neither in outpatient level nor inpatient level $P\left({y}_{1}=0\ and\ {y}_{2}=0\right),$ avoiding in outpatient level but not inpatient level $P\left({y}_{1}=1\ and\ {y}_{2}=0\right),$ not avoiding in outpatient level but inpatient level $P\left({y}_{1}=0\ and\ {y}_{2}=1\right),$ avoiding in both level$P({y}_{1}=1\ and\ {y}_{2}=1)$.

Table 3 shows that the coefficient of bivariate probit regression model in which the dependent variables avoidance attitudes ${y}_{1}$, ${y}_{2}$ and explanatory variables, which have been chosen by literature [1, 4, 12, 32, 42] are considered socio-economics factors. Here, age takes one if the individual’s age is 45 and over and zero otherwise; education level is takes one if individual’s education level is high school and over and zero otherwise. Household monthly variable is expressed in four categories: =0 if less or equal to 750 TL, = 1 if 751 TL to 1300TL, = 2 if 1301 TL to 2300TL and = 3 if greater than 2300TL; SSI takes one if the individual has social insurance or green card and zero otherwise, and persons to trust takes one if the number of people to trust greater or equal to two and zero if the number of people to trust is less or equal to one. Furthermore, we generated two indexes: index 1 and index 2, by considering the following three questions on well-being in 2012 Health Survey Data: “feel pain or discomfort in last four weeks”, “feel happy in last four weeks” and “perceived health”. The multiple-choice answers for the each of the questions were ‘all of the time’, ‘most of the time’, ‘some of the time’, ‘a little of the time’ and ‘none of the time’. The binary variable “Index 1” represents the best perceived state of the individuals which takes one if the respondent answers each of the three questions as ‘all of the time’ and zero otherwise. On the contrary, “Index 2” represents the worst perceived case of the individuals that takes one if the respondent answers each of the three questions as ‘none of the time’ and zero otherwise. BMI is the numerical value of the individual’s body mass index.

**Table 3:** Bivariate Probit Regression of Avoiding Outpatient and Inpatient Healthcare Services

Avoid, Outpatient					Avoid, Inpatient				
Variables	Coefficient	*p*	95% Confidence			Coefficient	*p*	95% Confidence	
Female	0.183	*p* < 0.001*	0.115	0.250	Female	0.165	0.004*	0.051	0.279
Age ≥ 45	-0.038	0.257	-0.105	0.028	Age ≥ 45	0.028	0.608	-0.080	0.136
Urban	-0.012	0.732	-0.083	0.058	Urban	-0.015	0.783	-0.126	0.095
Married	0.054	0.110	-0.012	0.119	Married	0.067	0.229	-0.042	0.176
Education level	-0.044	0.266	-0.121	0.033	Education level	-0.083	0.249	-0.225	0.058
Income (reference: Less or equal to 750 TL)					Income (reference: Less or equal to 750 TL)				
751 TL to 1300 TL	-0.189	*p* < 0.001*	-0.267	-0.110	751 TL to 1300 TL	-0.143	0.023*	-0.266	-0.020
1301 TL to 2300 TL	-0.243	*p* < 0.001*	-0.329	-0.157	1301 TL to 2300 TL	-0.211	0.004*	-0.352	-0.069
Greater than 2300 TL	-0.265	*p* < 0.001*	-0.370	-0.160	Greater than 2300 TL	-0.236	0.013*	-0.423	-0.049
Employed	0.206	*p* < 0.001*	0.134	0.278	Employed	0.086	0.172	-0.037	0.208
SSI	-0.350	*p* < 0.001*	-0.458	-0.243	SSI	-0.058	0.540	-0.244	0.128
Persons to trust ≥ 2	-0.166	*p* < 0.001*	-0.235	-0.096	Persons to trust ≥ 2	-0.109	0.049*	-0.217	0.000
Index 1	-0.463	*p* < 0.001*	-0.711	-0.215	Index 1	0.001	0.994	-0.360	0.363
Index 2	0.595	0.142	-0.199	1.390	Index 2	-4.594	*p* < 0.001*	-4.821	-4.367
BMI	-0.047	0.008*	-0.082	-0.012	BMI	-0.063	0.011*	-0.112	-0.015
Using non-prescription medications	0.445	*p* < 0.001*	0.364	0.525	Using non-prescription medications	0.291	*p* < 0.001*	0.166	0.416
Any-chronic	0.412	*p* < 0.001*	0.345	0.478	Any-chronic	0.562	*p* < 0.001*	0.443	0.680
Diabetes	-0.062	0.257	-0.170	0.045	Diabetes	-0.062	0.440	-0.218	0.095
Cancer	-0.178	0.297	-0.512	0.157	Cancer	0.086	0.712	-0.371	0.543
Constant	-0.816	*p* < 0.001*	-0.991	-0.641	Constant	-2.025	*p* < 0.001*	-2.300	-1.750

Note: *Significant at 5%

*athrho = 0.6418926 (p = 0.0000), 95% Confidence: [0.5708817–0.7129034]*

*rho = 0.5661868, 95% Confidence: [0.5160065–0.6124943]*

*n = 11,824, Log pseudolikelihood =-5652.9269, Wald chi*^*2*^ *= 2472.15, Prob > chi*^*2*^ *= 0.0000*

**Table 4:** Marginal Effects of Bivariate Probit Regression

Not Avoid Outpatient and Not Avoid Inpatient					Avoid Outpatient and Not Avoid Inpatient				
Variables	Coefficient	*p*	95% Confidence		Variables	Coefficient	*p*	95% Confidence	
Female	-0.040	*p* < 0.001*	-0.054	-0.026	Female	0.031	*p* < 0.001*	0.019	0.044
Age ≥ 45					Age ≥ 45				
Urban					Urban				
Married	-0.012	0.081*	-0.026	0.001	Married				
Education level					Education level				
Income (reference: Less or equal to 750 TL)					Income (reference: Less or equal to 750 TL)				
751 TL to 1300 TL	0.044	*p* < 0.001*	0.026	0.062	751 TL to 1300 TL	-0.035	*p* < 0.001*	-0.051	-0.019
1301 TL to 2300 TL	0.055	*p* < 0.001*	0.036	0.074	1301 TL to 2300 TL	-0.044	*p* < 0.001*	-0.061	-0.026
Greater than 2300 TL	0.060	*p* < 0.001*	0.038	0.082	Greater than 2300 TL	-0.047	*p* < 0.001*	-0.067	-0.027
Employed	-0.042	*p* < 0.001*	-0.057	-0.027	Employed	0.038	*p* < 0.001*	0.024	0.051
SSI	0.069	*p* < 0.001*	0.047	0.091	SSI	-0.066	*p* < 0.001*	-0.086	-0.046
Persons to trust ≥ 2	0.035	*p* < 0.001*	0.020	0.049	Persons to trust ≥ 2	-0.029	*p* < 0.001*	-0.042	-0.016
Index 1	0.089	0.001*	0.037	0.142	Index 1	-0.089	*p* < 0.001*	-0.134	-0.045
Index 2					Index 2	0.230	0.004*	0.075	0.384
BMI	0.011	0.003*	0.004	0.018	BMI	-0.008	0.024*	-0.014	-0.001
Using non-prescription medications	-0.093	*p* < 0.001*	-0.110	-0.077	Using non-prescription medications	0.079	*p* < 0.001*	0.064	0.094
Any-chronic	-0.094	*p* < 0.001*	-0.107	-0.080	Any-chronic	0.065	*p* < 0.001*	0.053	0.078
Diabetes					Diabetes				
Cancer					Cancer				

Note: *Significant at 5%

In Table 3, it can be seen that married and female ones are more likely to avoid heath care in both inpatient and outpatient health services, while people of having higher education level, living urban area and higher income level are less likely to avoid. Being + 45 age, best state of the individuals (index 1), worst state of the individuals (index 2) and having cancer have different effects on the avoidance of inpatient and outpatient health service. Individuals in these groups are less likely to avoid outpatient level and more likely to avoid inpatient level. In worst state of the individuals (index 2), the situation is the opposite. The higher number of people that individuals can trust and the higher BMI decrease the possibility of avoiding health services. However, participants having any-chronic disease and using non-prescription medications are more likely to delay or avoid inpatient and outpatient health services.

Table 4 shows the marginal effects of two joint probabilities^2^: First, not avoidance of outpatient and inpatient services; secondly, avoidance of outpatient services but not avoidance of inpatient services. In parallel with the expectations, the signs of joint probabilities are the opposite of each other. As seen in Table 4, if the individual is female, the probability of avoiding both outpatient and inpatient health services decreases by 4%. When the individual has any-chronic disease or using non-prescription medications, this probability increases by more than 9%. If the individual is female, the probability of avoiding both outpatient and inpatient health services decreases by 4%. Participants who are covered by social security institution -SSI- and green card- a public scheme for poor, the probability of avoidance of both outpatient and inpatient health services decreases by 6.9%.

## Discussion

Our study examines the factors affecting the avoidance of inpatient services in accordance with outpatient services in Türkiye, by using the 2012 Health Survey via bivariate probit model. Empirical literature generally has focused on developed country cases [1, 4, 7, 10, 11, 13, 18–20, 22, 28, 33, 34, 36, 37, 50–54] so we contribute to the knowledge considering a developing country case.

According to our descriptive statistics, analysis 2.6% of the participants did not received deemed necessary inpatient services, and avoiders were more likely to be female, married, uninsured, middle aged or older, low-educated, living in rural area and having lower income, chronic disease and use non-prescription medications. Hence, we found that disadvantaged groups tend to avoiding necessary healthcare services, therefore the hypothesis of our study was met. Moreover, it is found that high cost (31%), organizational factors (21%) and fear (12%) are main reasons of avoiding. Similar to our findings, “money restriction” was the first avoiding reason in[42]; but [4, 11, 36] indicate that “fear” was one of the most important reasons.

Mostly, our bivariate probit model findings were in line with the literature. Similar to [1, 55] an important finding of our analysis was that the ones who have lower income level are more likely to avoid. Although [4, 9, 12] found evidence that being male was related to health care avoidance, we found this relation for being female. As oppose to [4] the evidence on that BMI is associated with greater avoidance; we observed that the higher BMI decreased the possibility of avoiding health services. Similar to [12], we found a negative relationship between living urban area and health care avoidance. Furthermore, our study finds out that perceived health affects differently people’s avoidance attitude at different levels of healthcare. Even though people with the worst perceived health intend to avoid healthcare in outpatient level, they behave less likely to avoid in inpatient level. Even this attitude underlines that the avoided healthcare needs may require more serious and costly treatment.

### Limitations

There are some limitations in this study. We used TurkStat’s 2012 Health Survey Data that has similar limits of cross-sectional data as stated in [15]. Firstly, since it is not panel data, we cannot eliminate the unobserved bias of the individuals. Another limitation is the structure of question on reasons of avoidance similar to the study of [1], participants were asked to choose strictly only one among eight pre-defined reasons. However, in reality, people may have more than one reasons of avoidance. In this situation, while the most common reason comes to the forefront, prevalence of all reasons may be underestimated. In addition, reasons stated in our 2012 Health Survey Data were qualitative, that may create difficulties in measuring patterns of avoidance, and thus using quantitative like Likert scale would be more reliable as suggested in [1].

Moreover, in our study, we only investigated the avoided need of healthcare since our data was only querying the avoided needs. Actually, unmet health needs are a comprehensive concept, and avoiding healthcare needs is only a part of it, but for our sake we should underline that still avoided healthcare needs constitute a significant part of the unmet needs [56].

## Conclusion

We found that high cost, organizational factors and fear were the main reasons of avoiding inpatient health services in accordance with outpatient services. We found that, in both stages of healthcare, avoiders were more likely to be female, married, middle aged or older, low-educated, living in rural area and having lower income, chronic disease and used non-prescription medications.

In the light of our findings, we recommend policy makers to improve health care protection of the disadvantaged groups and develop better organizational factors to prevent difficulty of having treatment at policlinics. Finally, as there is a limited number of studies especially on developing countries, we should indicate that further studies are required to distinguish people’s avoidance attitude at different stages of health care utilization. Indeed, the influence of COVID19 pandemic on avoidance behavior appears as crucial topic to search.

## Data Availability

TurkStat prohibits the sharing of the data with the third parties, thus the authors cannot share it with the third parties. The TurkStat 2012 Health Survey Data cannot be copied or released to any other person or organization, as stated in the Article 14 of the Statistics Law of Türkiye No. 5429. However, all the datasets are available from corresponding author on reasonable request with permission from the third parties.

## Footnotes

1 Since missing observations have very small share (0.29%), we simply ignore them.

2 The reason for choosing these two joint possibilities ($P\left({y}_{1}=0\ \text{a}\text{n}\text{d}\ {y}_{2}=0\right), P\left({y}_{1}=1\ \text{a}\text{n}\text{d}\ {y}_{2}=0\right)$) is that their coefficients are approximately 10 times higher than other two joint possibilities ($P\left({y}_{1}=0\ \text{a}\text{n}\text{d}\ {y}_{2}=1\right), P({y}_{1}=1\ \text{a}\text{n}\text{d}\ {y}_{2}=1)$). The other two joint probabilities are available upon request.
